# The Risk of Cardiovascular Disease Among Immigrants in Canada

**DOI:** 10.7759/cureus.22300

**Published:** 2022-02-16

**Authors:** Sneha A Sebastian, Chaithanya Avanthika, Sharan Jhaveri, Keila G Carrera, Genesis P Camacho L, Ramya Balasubramanian

**Affiliations:** 1 Medicine and Surgery, Azeezia Medical College, Kollam, IND; 2 Medicine and Surgery, Karnataka Institute of Medical Sciences, Hubli, IND; 3 Medicine and Surgery, Nathiba Hargovandas Lakhmichand Municipal Medical College (NHLMMC), Ahmedabad, IND; 4 Gastroenterology, Universidad de Oriente - Venezuela, Maturin, VEN; 5 División de estudios para graduados, Facultad de Medicina., Universidad del Zulia, Maracaibo, VEN; 6 Pediatrics, Smt Kashibai Navale Medical College, Pune, IND

**Keywords:** immigrants in canada, ethnicity, cvd risk among immigrants, cvd risk factors, cardiovascular disease

## Abstract

Cardiovascular disease (CVD) is the leading cause of morbidity and mortality worldwide. The global surge in migration to high-income countries, especially Canada, highlights the importance of studies evaluating the risk factors and the disparities in the rate of incidence of CVD among immigrants. Canada is home to a diverse group of immigrants, each presenting with a risk profile that is unique to their ethnicity and country of birth. A variety of cardiac risk factors, such as dietary habits, physical activity, smoking, cultural traditions as well as preponderance to certain diseases like type II diabetes mellitus, hypertension, and high lipid levels act in concert and impact CVD risk and overall incidence. This narrative review focuses on CVD risks and how it is related to the immigration status among various ethnic groups in Canada.

## Introduction and background

Cardiovascular disease (CVD) risks are of concern among immigrants and refugees settling in developed host countries [1, 2]. With nearly 7.5 million persons moving to Canada through immigration, Canada has one of the highest per-capita immigration rates of any high-income country, accounting for almost one-fifth (21.9%) of the total population [3]. According to demographic forecasts from Statistics Canada, the proportion of foreign-born Canadians might reach 24.5% to 30.0% by 2036 [3]. Among the immigrants, South Asians are the single largest visible minority group, accounting for 29.6% of visible minorities in Canada [3, 4]. Various factors like socioeconomic status, education and healthcare behaviors, and attitudes among these immigrant populations can significantly impact their risk, treatment, and outcomes of atherosclerotic cardiovascular disease (ASCVD) [5]. Also, most of the immigrants in Canada are from low and middle-income countries having the highest incidence of cardiovascular deaths [6].

As per studies, there are eight major ethnic groups in Canada defined based on certain shared characteristics, including geographical and ancestral origins, particularly cultural traditions. And there is a significant variation in the incidence of CVD based on ethnicity and country of origin [7, 8]. This necessitates the importance of research studies exploring the CVD risk factors among immigrants based on ethnicity and country of birth. In this review, we will discuss the burden of cardiovascular risk factors and their relation to the immigration status among various ethnic groups in Canada.

## Review

We did a literature search of relevant articles over the last two decades from the databases namely PubMed and Google Scholar using the following keywords; "Cardiovascular disease", "CVD risk factors", "CVD risk among immigrants", "ethnicity", and "Immigrants in Canada" until Jan 24, 2022. For the review, inclusion criteria comprised of studies published in the English language irrespective of study design that evaluated the risk of CVD among immigrants in Canada. We excluded articles published in other languages.

Incidence and demographics

The burden of CVD among immigrants in Canada varies based on country of birth and ethnicity. Based on the Cardiovascular Health in Ambulatory Care Research Team (CANHEART) immigrant study conducted in 2015, including a cohort of 824, 662 first-generation immigrants linking information from Citizenship and Immigration Canada's Permanent Resident database to nine population-based health databases, the overall incidence of major cardiovascular events was 30% lower in recent immigrants than in long-term residents [8]. Among them, male immigrants had a greater cardiovascular event rate (30%) compared to females (29%) [8].

Considering various ethnic groups in Canada, immigrant South Asians had a higher incidence of acute myocardial infarction (MI) compared to white people: 4.97 events per 1000 population per year among South Asian men versus 3.29 among white men; 2.35 events per 1000 population per year among South Asian women versus 1.53 among white women [8, 9].

The prevalence of CVD, which includes a spectrum of the history of MI, angina, silent MI, percutaneous transluminal coronary angioplasty, coronary artery bypass grafting, or stroke, was higher among South Asians: 5.7% to 10.0% versus 5.4% to 5.7% among white people. Mortality rates from coronary heart disease were also higher among South Asians, with the data collated from 1979 to 1993, 42% in men versus 29% in women [9]. All the data was found to be statistically significant. Also, South Asians living in Canada have a higher prevalence of hypertension, type II diabetes mellitus, and insulin resistance. They had a higher percentage of body fat, visceral adiposity, a higher ratio of apolipoprotein B to apolipoprotein A-1, higher carbohydrate intake, lower high-density lipoprotein (HDL) cholesterol levels, and lower levels of physical activity, which contributed to a higher cardiovascular event rate in this group [9].

Risk factors contributing to the increased risk of cardiovascular disease in immigrants

Most of the risk factors for the development of CVD are shared by the general population; however, some stand out, particularly among various ethnic groups. Smoking, high blood pressure, type II diabetes mellitus, and high cholesterol levels are among the modifiable factors, with the latter being largely associated with dietary habits. South Asians are the migrant group in North America with the highest cardiovascular risk of any ethnic group [8, 10, 11]. One of the reasons is the adaptations in the dietary pattern of first-generation and successive generations of South Asians who immigrated to Canada. Consumption of a diet high in carbohydrates, low in fruits and vegetables, and high salt intake lead to an increase in lipid levels and, as a result, an increase in abdominal circumference. Dietary habits combined with other traditional risk factors make them more prone to CVD [11]. Also, the high prevalence of insulin resistance and type 2 diabetes mellitus in Asians may be a major cause of their elevated vascular risk [12]. An important phenomenon among this group is the belief that larger bodies directly translate into better health, stronger children, and better nutrition. Furthermore, it is noteworthy that South Asians do not engage in physical activity regularly, let alone when it is associated with health promotion. Finally, South Asians are resistant to any changes in their lifestyle and diet that they believe will have an impact on their cultural identity [8, 10, 11].

Concerning the level of knowledge about CVD risk factors, Langellier et al. using the National Health and Nutrition Survey from 2003 to 2008, found that, in general, the entire population does not have a good level of awareness about CVD and its risk factors; however, the higher prevalence is among the immigrant population, particularly those who do not speak English or speak English as a second language, making the language barrier an impediment [13]. Health education and access to healthcare services also have an impact on the routine screening and care of chronic medical conditions like type II diabetes mellitus, hypertension, and high cholesterol, the modifiable CVD risk factors, among those with low income or who are considered illegal immigrants. [13, 14].

An immigrant cohort study conducted by Tu et al. in 2015 from Ontario, Canada, revealed that in general, the rate of occurrence of major cardiovascular events in immigrants was 30 percent lower compared to the long-term residents. However, there were variations among this rate in different ethnic groups in which the highest CVD risk was in South Asians and the least CVD risk among East Asians [8]. Their study concluded that the incidence of cardiovascular events was similar in most ethnic groups while comparing the duration of residence in Canada for less than ten years against more than ten years [8].

The risk factors for CVD may differ between immigrant groups. The average might be higher or lower depending on different factors such as the country of origin of immigrants, and this is due to their habits and culture especially, the prevalence of psychological stress and mental health issues among different ethnic groups. Mental health issues are relatively predominant among South Asians because of the social stigma associated with this topic that hinders them from seeking help. Country to which people emigrate also has a vital role in the incidence of CVD due to the cultural changes, mainly the biological interactions between genetic and environmental factors [8, 15].

Cardiovascular disease risk factors by ethnicity

CVD and its risk factors are heading on the list of preventable causes of morbidity and mortality in Canada. Ethnicity is considered a relevant component when discussing the CVD risk factors because of drastic changes immigrants are exposed to when arriving in developed countries such as the US and Canada, where the life rhythm and food habits are distant by culture. Also, change in the level of physical activity influenced by the living conditions and socioeconomic status and access to healthcare services increase CVD risk [1].

Although, the incidence of CVD is dependent on many factors such as genetics, environment, host country, and food habits. Many research studies have found out that there is an increase in the rate of CVD risk factors by country of origin. Canada’s demographic composition is ethnically heterogeneous and based on country of birth, mother tongue, and surname, there are eight major ethnic groups in Canada: (1) East Asian (e.g. Chinese, Korean); (2) Southeast Asian (e.g. Filipino, Vietnamese); (3) Black, from Sub-Saharan Africa and the Caribbean; (4) West Asian/Arab, from the Middle East, West Asia, and some former republics of the Union of Soviet Socialist Republics (USSR); (5) Latin American, from Central and South America; (6) South Asian (e.g. Pakistani, Indian, Sri Lankan); and white immigrants subdivided into those of (7) White Eastern European and (8) White Western European origin (e.g. the United States, Australia, New Zealand, and South Africa) [8]. CVD risk factors vary among different ethnic groups, and the prevalence of common modifiable risk factors differs between males and females of each ethnic group [8].

Asian Immigrants

In 2014, Rana et al. demonstrated by their analysis that Asian immigrants specifically, South Asians, have a high risk of CVD (5.7%-10%) compared with the white Canadians (95.4%-5.7%) [9]. With regards to CVD risk factors, Asian immigrants have increased risk factors relative to white Canadians. Among these are hypertension {odds ratio [OR] 1.11, 95% confidence interval [CI] = 1.02-1.2, (p) = 0.02}; type II diabetes mellitus {OR 2.25, 95% CI = 1.81-2.8, (p) < 0.001} [9]. However, obesity [Body mass index (BMI) > 30] and smoking were less in South Asian immigrants {OR 0.38, 95% CI = 0.24-0.6, (p) < 0.001} compared to white Canadians {OR 0.62, 95% CI = 0.40-0.96, (p) = 0.03} [9]. Furthermore, Jeemon et al. in 2009 demonstrated that Indian immigrants tend to have premature CVD at least a decade before compared with white Canadians [16]. These results evidence the clear clinical importance of risk factors in Asian immigrants that moved to developed countries such as Canada, and their risk for CVD.

On the other hand, Chinese immigrants were less likely to have CVD (OR 0.44, 95% CI = 0.24-0.94), because they had lower BMI, lower average sex-adjusted low-density lipoprotein (LDL) cholesterol, and higher average sex-adjusted HDL cholesterol. However, the prevalence of type II diabetes mellitus and hypertension was similar to white Americans [16, 17]. Based on Chiu et al.'s multi-ethnic study using the data from Statistics Canada's National Population and Canadian Community Health Surveys from 1996 to 2007, Chinese immigrants had a greater risk of CVD based on the duration of residence in Canada. Chinese immigrants with more than 15 years of stay in Canada (long-term residents) reported having 5.2% CVD, and recent Chinese immigrants with less than 15 years of residence in Canada had only 2.2% CVD [18].

African Immigrants

Among Somali immigrants and refugees, there is a high-level prevalence of obesity, type II diabetes mellitus, and low physical activity that puts them at risk for CVD, mainly they are affected by the cultural barriers, rate of health insurance, and established primary care. Overall, the CVD risk was higher among males than females in this immigrant group [1].

Black immigrants living in Ontario, Canada tend to have more prevalent risk factors of CVD such as type II diabetes mellitus (27.6% male and 94.2% women) vs white Canadians (18.8% male and 21.7% women). The prevalence of other risk factors among black immigrants in Ontario was current smoking status (7.2% male and 11.6 women) and hypertension (8.3% male and 30% women). This shows a significant impact on having CVD earlier than other ethnicities [19]. Black immigrants also tend to have high rates of stroke and acute MI, which is attributed to a high rate of hypertension, high smoking rate, high levels of HDL, and low fibrinogen levels [8].

In 2019, Di Giuseppe et al. reported that the incidence of heart failure (HF) hospitalization was high in Black men (1.19 per 1000 person-years) based on the findings of a retrospective observational study from 2008 to 2012, including 895, 823 recent immigrants from eight ethnic groups and 5.3 million long-term residents aged 40 to 105 years. This study highlighted the importance of considering ethnicity as a potential independent risk factor for CVD [20].

European Immigrants

Studies found that Eastern European immigrants have a greater likelihood of experiencing CVD and stroke relative to white Canadians. The duration of residence in Canada has increased their risk for stroke and CVD [21]. Among their risk factors, the smoking rate was high in males and females. Obesity, type II diabetes mellitus, and hypertension were low compared to white Canadians [8].

Hispanic Immigrants

Liu et al. using three cross-sectional analyses of the Canadian Community Health Survey from 2000, 2003, and 2005 reported that people of the Hispanic ethnic group were physically inactive as the white population, which has contributed to their CVD risk [22]. Cigarette smoking, one of the common modifiable CVD risk factors, was high among Hispanic males compared to females. The proportion of Hispanic males and females affected by hypertension and type II diabetes mellitus was high relative to white Canadians [8]. Table 1 summarizes the prevalence of common CVD risk factors among immigrants in Canada based on ethnicity.

**Table 1:** Summary of the prevalence of common CVD risk factors among immigrants in Canada. *Prevalence of CVD risk factors in descending order, CVD: Cardiovascular disease, Tu JV, Chu A, Rezai MR, et al. The CANHEART Immigrant Study [8].

Ethnic Group	Prevalence of CVD Risk Factors Among Males Based on Ethnicity*	Prevalence of CVD Risk Factors Among Females Based on Ethnicity*
East Asian	Cigarette smoking > hypertension > type II diabetes mellitus > high cholesterol level > obesity	Hypertension > high cholesterol level > type II diabetes mellitus > obesity > cigarette smoking
Black	Hypertension > cigarette smoking > obesity > type II diabetes mellitus > high cholesterol level	Hypertension > obesity > type II diabetes mellitus > high cholesterol level > cigarette smoking
South Asian	Hypertension > cigarette smoking > type II diabetes mellitus > obesity > high cholesterol level	Hypertension > type II diabetes mellitus > obesity > high cholesterol level > cigarette smoking
Latin American	Cigarette smoking > hypertension > obesity > type II diabetes mellitus > high cholesterol level	Hypertension > obesity > type II diabetes mellitus > cigarette smoking > high cholesterol level
White-Western European	Cigarette smoking > obesity > hypertension > high cholesterol level > type II diabetes mellitus	Obesity > hypertension > cigarette smoking > type II diabetes mellitus > high cholesterol level

The pattern of incidence of cardiovascular events by country of origin

There are noticeable differences in the rate of occurrence of cardiovascular events among immigrants in Canada based on their country of origin. The CANHEART immigrant study conducted in 2002 based on a cohort of 824, 662 first-generation immigrants from eight major ethnic groups namely East Asian, Southeast Asian, Blacks, West Asian/Arab, Latin American, white Eastern European, and white Western European origin, and 201 countries of birth, reported that the incidence of cardiovascular events was similar among different countries of origin within each geographical region of origin, however, few exceptions were noted [8]. Immigrants from Iraq and Afghanistan had higher cardiovascular event rates than those born in neighboring countries namely Lebanon, Iran and Egypt. Immigrants from Iraq and Afghanistan also had the highest cardiac risk factor score which is a point-based score to quantify the burden of four modifiable risk factors like smoking, type II diabetes mellitus, hypertension, and high lipid levels [8, 23]. Among these immigrants, refugees had the highest proportion of cardiovascular risk factor scores. Immigrants from Iraq and Afghanistan had a cardiac risk factor score of 61% and 71 % respectively relative to other immigrants from the Middle East and West Asia which were only 19%. Immigrants born in Guyana and Trinidad and Tobago also had a high incidence of cardiovascular events [8].

Overall, South Asians are reported to have the greatest risk of cardiovascular events among immigrants of Canada. Sri Lankan males and Bangladeshi females had the highest proportion of cardiovascular events among them. East Asians had the least risk of CVD, especially people born in Taiwan and later migrated to Canada. Even though the rate of incidence of cardiovascular events is comparatively low among Latin Americans, Guyanese people showed a disproportionate increase in the rate of cardiovascular events similar to South Asians [8]. Table 2 details the incidence of cardiovascular events by country of birth based on the findings from the CANHEART immigrant study by Tu et al.

**Table 2:** Incidence of cardiovascular events among immigrants in Canada by country of birth. *Incidence rate in descending order. Tu JV, Chu A, Rezai MR, et al. The CANHEART Immigrant Study [8].

Geographical Region	Countries	Incidence of Cardiovascular Events Among Males by Country of Birth *	Incidence of Cardiovascular Events Among Females by Country of Birth *
East Asia	Taiwan, Hong Kong, China, South Korea	South Korea > China > Hong Kong > Taiwan	South Korea > China > Hong Kong > Taiwan
Western Europe/United States	The United States, Portugal, The United Kingdom	The United Kingdom > Portugal > The United States	The United Kingdom > Portugal > The United States
Sub-Saharan Africa/Caribbean	Ghana, Somalia, Jamaica, Ethiopia, Trinidad & Tobago	Trinidad & Tobago > Jamaica > Ethiopia > Somalia > Ghana	Trinidad & Tobago > Jamaica > Ghana > Somalia > Ethiopia
Southeast Asia	Vietnam, Philippines	Philippines > Vietnam	Philippines > Vietnam
Latin America	El Salvador, Guyana	Guyana > El Salvador	Guyana > El Salvador
The Middle East/West Asia	Iran, Lebanon, Iraq, Afghanistan, Egypt	Iraq > Afghanistan > Egypt > Lebanon > Iran	Afghanistan > Iraq > Egypt > Iran > Lebanon
Eastern Europe	Romania, Yugoslavia, Poland, Russia	Poland > Russia > Yugoslavia > Romania	Poland > Russia > Yugoslavia > Romania
South Asia	India, Bangladesh, Sri Lanka, Pakistan	Sri Lanka > Pakistan > Bangladesh > India	Bangladesh > Pakistan > India > Sri Lanka

## Conclusions

The impact of CVD on different ethnic groups varies as each group has a unique risk profile. There is a striking difference in the incidence of cardiovascular events based on ethnicity and country of origin. Also, the prevalence of common modifiable cardiac risk factors including, smoking, hypertension, type II diabetes mellitus, and high cholesterol levels, differ based on ethnicity. Early identification and management of the traditional cardiac risk factors among each ethnic group combined with strategies to address the CVD risk factor unawareness and disparities in access to healthcare services can reduce CVD morbidity and mortality among immigrants. Through this review, we aim to provide some insight into the burden of cardiovascular events and their associated risk factors among immigrants in Canada. We also hope to further scientific discovery in this relatively underexplored subject to improve cardiac health outcomes among newcomers to Canada.
